# Point-of-care troponin tests to rule out acute myocardial infarction in the prehospital environment: a protocol for a systematic review and meta-analysis

**DOI:** 10.1136/bmjopen-2024-094390

**Published:** 2025-05-02

**Authors:** Bader Albaqami, Jacqueline Dinnes, Theresa HM Moore, Kim Kirby, Simon D Carley, Mohammad Aloufi, Naif Alqurashi, Abdulrhman Alghamdi, Sara Alsuwais, Sarah Dawson, Richard Body

**Affiliations:** 1 Division of Cardiovascular Sciences, The University of Manchester, Manchester, UK; 2 University of Bisha, Bisha, Saudi Arabia; 3 Department of Applied Health Sciences, College of Medicine and Health, University of Birmingham, Birmingham, UK; 4 NIHR Birmingham Biomedical Research Centre, University Hospitals Birmingham NHS Foundation Trust and University of Birmingham, Birmingham, UK; 5 Population Health Sciences, NIHR CLAHRC West, Bristol, UK; 6 NIHR CLAHRC West, University of Bristol, Bristol, UK; 7 University of the West of England, Bristol, UK; 8 The University of Manchester, Manchester, UK; 9 University of Bisha, Bisha, Asir, Saudi Arabia; 10 School of Medical Sciences, The University of Manchester, Manchester, UK; 11 Accidents and Trauma Department, Prince Sultan Bin Abdulaziz College for Emergency Medical Services, King Saud University, Riyadh, Saudi Arabia; 12 King Abdullah International Medical Research Center, Riyadh, Saudi Arabia; 13 Department of Emergency Medical Services, College of Applied Medical Sciences, King Saud Bin Abdulaziz University for Health Sciences, Riyadh, Saudi Arabia; 14 ARC West, NIHR ARC West, Bristol, UK; 15 Populations Health Sciences, University of Bristol Medical School, Bristol, UK; 16 Division of Cardiovascular Sciences, University of Manchester, Manchester, UK

**Keywords:** Coronary intervention, Cardiovascular Disease, Emergency Service, Hospital, Cardiology, Myocardial infarction

## Abstract

**Abstract:**

**Background:**

Chest pain is a major cause of emergency ambulance calls, often linked to acute myocardial infarction (AMI), a critical condition requiring immediate hospitalisation. Current diagnostic methods, such as history taking and ECG, have limitations, especially for non-ST-elevation myocardial infarction. High-sensitivity cardiac troponin (cTn) assays are more diagnostically sensitive, but the downside is that it needs hospital-based testing, which can delay diagnosis and the necessary treatment protocol. Point-of-care cTn testing, on the other hand, is much faster and done nearer to the patient; hence, it may fundamentally change the prehospital care pathway in terms of diagnostic accuracy, clinical utility and related safety.

**Objective:**

To present a protocol for a systematic review and meta-analysis that will assess the diagnostic accuracy, clinical utility and safety of point of care (POC) troponin tests, with or without clinical decision aids, for ruling out AMI in adults presenting with cardiac chest pain to emergency ambulance services in prehospital settings.

**Methods:**

This protocol follows BMJ guidelines and adheres to the Preferred Reporting Items for Systematic Review and Meta-Analysis Protocols 2015 reporting standards. It is registered with PROSPERO (ID: CRD42024533117). A comprehensive search strategy will identify relevant studies in MEDLINE, EMBASE and CINAHL, focusing on literature from 2000 onwards. Eligibility criteria include adults with chest pain suspected of AMI, excluding those with ST-elevation myocardial infarction. The primary target is type 1 AMI, with secondary outcomes including major adverse cardiac events at 30 days. Risk of bias assessment will be performed using tools such as Quality Assessment of Diagnostic Accuracy Studies version 2, Risk of Bias 2, and Risk of Bias in Non-randomised Studies of Interventions, while the quality of the economic evaluations will be appraised using the Consolidated Health Economic Evaluation Reporting Standards (CHEERS) checklist. Data items extracted will include patient demographics, test characteristics and outcomes. Where possible, meta-analyses will be conducted by fitting hierarchical models for diagnostic accuracy and random effects models for clinical and cost-effectiveness estimates. Subgroup analyses are proposed to quantify the effect of variables such as gender, ethnicity and type of troponin assay on the estimated parameters.

**Ethics and dissemination:**

Ethical approval is not required. The results will be published in a peer-reviewed journal and presented at international conferences.

**PROSPERO registration number:**

This protocol is registered with PROSPERO, the International Prospective Register of Systematic Reviews, under the ID CRD42024533117. Any future amendments will be updated in the PROSPERO record.

**STRENGTHS AND LIMITATIONS OF THIS STUDY**

- Follows Preferred Reporting Items for Systematic Review and Meta-Analysis Protocols/Cochrane guidelines: PROSPERO-registered (CRD42024533117) for transparency. Variability in prehospital protocols (eg, assay thresholds and HEART/T-MACS (Troponin-only Manchester Acute Coronary Syndromes) decision aids) may limit comparability.
- Focuses on paramedic-led diagnostics and evaluates accuracy and clinical outcomes but may lack power for rare events (eg, 30-day major adverse cardiac event).
- Prioritises post-2000 evidence and captures high-sensitivity troponin advances, though rapid assay advancements may outdate older studies.
- Uses Quality Assessment of Diagnostic Accuracy Studies version 2/Risk of Bias 2 tools and assesses bias rigorously but risks verification bias from hospital-based adjudication in non-transported patients.
- Includes translated non-English studies and reduces geographic bias; translation challenges may affect data accuracy.

## Introduction

 Chest pain is one of the most common reasons for calls to Emergency Medical Services (EMS), which places immense pressure on the healthcare system. In England, chest pain accounts for about 2000 calls a day.^1^ Patients suffering from chest pain may be experiencing an acute myocardial infarction (AMI). AMI is a potentially life-threatening condition, and the patient needs to be hospitalised immediately. The current approach to diagnosing an AMI in the EMS setting is history taking and ECG, but these have certain limitations. While an ECG can be used in the detection of ST-elevation myocardial infarction, where there is a major artery blockage, it is less definitive for non-ST-elevation myocardial infarction (NSTEMI), which is more common but equally severe.^2^ Thus, the majority of cases will require troponin testing in hospital settings, which in turn would have a lengthening diagnosis and treatment time.^3^

Cardiac troponin (cTn) is one of the cardiac muscle proteins that acts as a clinical biomarker for the diagnosis of AMI. In ordinary conditions, the level of this protein in the blood would be virtually undetectable; however, in cases of injury to the heart muscle, these levels will be significantly higher.^4^ High-sensitivity troponin assays have therefore brought in much improvement in diagnostic sensitivity, thus allowing the use of a rapid rule-in/rule-out algorithm for the presence of AMI directly in emergency departments.^5^ Nevertheless, the standard approach to its use includes the need to transport the patient to hospital for a troponin blood test and central processing in a hospital laboratory, thus delaying patient management.^4 6^ These delays not only extend the time paramedics spend on each case but also reduce the availability of ambulance services for other emergencies. Additionally, longer diagnostic timelines can increase healthcare costs through prolonged ambulance activation, extended hospital stays and the need for additional diagnostic procedures, placing further strain on already stretched healthcare resources.^6 7^

CTn assays detect myocardial injury, but myocardial infarction (MI) diagnosis requires clinical evidence of ischaemia (eg, symptoms and ECG changes) alongside cTn elevation, as outlined in the Fourth Universal Definition of MI.^8^ While cTn cannot differentiate type 1 MI (atherosclerotic plaque rupture) from type 2 MI (ischaemia from increased oxygen demand or reduced supply), this protocol emphasises type 1 MI due to its relevance to prehospital interventions.^9^ Atypical symptoms such as dyspnoea or nausea occur in 20%–40% of MI cases, with higher rates in women, older adults and individuals with diabetes.^10^

The introduction of POC troponin testing into the prehospital setting has the potential to streamline patient pathways in the management of suspected AMI. Such POC tests allow near-patient testing with a result available within minutes, thus enabling the prehospital care pathway to be changed and reducing time to diagnosis.^11^ Evidence from a number of studies suggests that the development of POC troponin tests could maintain equivalent diagnostic accuracy to that of laboratory-based tests, especially in the recognition of NSTEMI.^12^ High-sensitivity POC cTn assays, such as the Roche cobas h232 troponin T and Siemens Atellica VTLi troponin I, are validated for clinical use. These assays meet ESC/ACC criteria for high sensitivity (≤10% coefficient of variation at the 99th percentile upper reference limit) and enable quantification of cTn concentrations as low as 3–5 ng/L.^7 8^ Studies demonstrate their diagnostic accuracy in prehospital settings, with performance comparable to laboratory-based assays.

This systematic review will guide EMS protocols for safely ruling out AMI before hospital arrival. Demonstrating the reliability of POC troponin assays could allow paramedics to safely avoid transporting low-risk patients and expedite treatment for those requiring urgent care.^1 13^ It may also help reduce unnecessary heparin use in non-ischaemic cases, improving patient safety and avoiding potential bleeding complications (14). These improvements may enhance ambulance service efficiency, lower healthcare costs and maintain patient safety.^3^ POC troponin testing in prehospital settings may reduce time to treatment, enabling faster initiation of therapies. This could improve survival, reduce complications and streamline care by ensuring timely intervention for high-risk patients and avoiding unnecessary hospital admissions for low-risk cases.^14^

Recent developments include clinical decision aids such as the HEART score and T-MACS, which combine troponin levels with clinical factors like patient history, symptoms and ECG findings. These tools help paramedics assess AMI risk more accurately, improving decision-making in prehospital settings and ensuring timely care for patients who need urgent intervention.^11 13^

A systematic review by Alghamdi *et al* highlighted the diagnostic potential of prehospital POC troponin tests in identifying AMI.^14^ However, the review identified gaps in the diagnostic accuracy of these tests, particularly in their ability to reliably rule out AMI before hospital admission. Studies reported widely varying sensitivity rates for POC troponin tests in detecting AMI, ranging from 26.5% to 91%. These inconsistencies likely arose from differences in troponin cut-off values and the lack of agreed on protocols for testing in ambulances. Many studies also suffered from small participant groups, poorly explained methods for confirming AMI diagnoses and no blinding of result factors that could bias outcomes and make findings hard to apply broadly. To address these gaps, future research should prioritise more precise assays, uniform cut-off standards and rigorously designed studies to confirm POC troponin’s reliability for diagnosing AMI outside hospitals.^14^

The rapid advancements in technology and the need for more efficient prehospital care pathways emphasise the necessity for an updated review. Significant advancements in high-sensitivity cTn assays since 2000 have improved analytical precision and clinical utility. Lower detection thresholds and integration with clinical decision tools have enhanced diagnostic accuracy, justifying this updated review to capture current evidence and address gaps highlighted by Alghamdi *et al*, ensuring relevance to contemporary prehospital care practices. The current technology offers improved sensitivity and faster results,^6^ which, when integrated with advanced clinical decision aids, can noticeably enhance early AMI detection and patient outcomes. Updating the systematic review is necessary to reflect these technological advancements and ensure that the most current and effective practices are employed in prehospital settings.

Recent systematic reviews have advanced understanding of troponin-based strategies for AMI rule-out. For instance, Westwood *et al* evaluated the clinical and cost-effectiveness of high-sensitivity cTn assays in hospital settings, demonstrating strong diagnostic accuracy for single and serial testing protocols. Their analysis emphasised that assays using thresholds near the limit of detection achieved high sensitivity, while repeat testing within 1–3 hours improved diagnostic confidence.^15^ The review also identified specific assays, such as those by Beckman Coulter, Roche and Siemens Healthcare, as cost-effective under healthcare budgets with defined willingness-to-pay thresholds. However, this work was confined to hospital environments, where delays in patient triage and laboratory processing persist.

In contrast, our systematic review shifts focus to POC troponin testing in prehospital settings. Prehospital care presents distinct challenges: paramedics operate with limited resources, face time-critical decision-making and manage delays inherent to patient transport. By evaluating the diagnostic accuracy, clinical utility and safety of POC troponin tests in EMS, this review addresses a critical gap in evidence. Furthermore, integrating POC testing with validated decision aids (eg, HEART score and T-MACS) could refine prehospital pathways, reduce unnecessary hospital admissions and expedite treatment factors unexplored in prior hospital-centric analyses.^11 13^

### Article summary

This proposed meta-analysis study design aims to build on previous work by evaluating the diagnostic accuracy, clinical utility and safety of POC troponin tests in prehospital settings, providing an updated synthesis of evidence to guide clinical practice.

### Objectives

This document presents a protocol for a systematic review and meta-analysis designed to assess the diagnostic accuracy, clinical utility and safety of POC troponin tests, with or without clinical decision aids, for ruling out AMI in adults presenting with cardiac chest pain to emergency ambulance services in prehospital settings.

## Methods

This systematic review protocol follows Preferred Reporting Items for Systematic Review and Meta-Analysis Protocols (PRISMA-P) guidelines to ensure clarity and transparency. Given the focus on diagnostic accuracy, the methods are also informed by the *Cochrane Handbook for Systematic Reviews of Diagnostic Test Accuracy*. A completed PRISMA-P checklist is provided as online supplemental file 2.

### Eligibility criteria

#### Diagnostic test accuracy

The eligibility criteria for studies evaluating the diagnostic test accuracy of POC troponin tests are essential for determining how well these tests perform in identifying or ruling out AMI in the prehospital setting. The specific criteria for these studies, including details on the patient population, index tests, comparators and reference standards, are summarised in table 1.

**Table 1:** PICTR 1: diagnostic test accuracy

Population	Adult patients (aged 18 years and over) receiving an emergency ambulance response for pain or discomfort in the chest, epigastrium, neck, jaw, arm(s) and/or throat that the treating paramedic suspects may have been caused by AMI.Exclusions: patients with ST-elevation myocardial infarction
Index test(s)	cTn point-of-care test +/− a clinical decision aid such as the HEART score or T-MACS decision aid, used to identify ‘very low risk’ patients who may avoid conveyance to hospital. Any POC troponin assay and any decision aid will be eligible. POC tests are defined as any test that can be administered and interpreted outside of a laboratory setting and within the confines of a single clinical encounter.We will include data for all cTn tests, but we will run subgroup analyses for high-sensitivity and contemporary troponin assays.Thresholds for test positivity: limit of detection of the assay; 99th percentile upper reference limit. We will accept thresholds as defined and reported in the included studies.Thresholds for decision aids: ‘very low risk’ for T-MACS; ‘low risk’ for HEART score; the lowest risk group for all other decision aids analysed.Blood samples should have been drawn in the prehospital environment. Tests should preferably be run by paramedics in the prehospital environment using whole blood samples; however, studies where the test was performed in a different setting will also be accepted and investigated as a potential source of heterogeneity in the analyses.
Comparator	Within-study direct comparisons between POC troponin assays, decision aids and the use of troponin alone vs decision aids will be included. Studies without direct comparators will also be accepted and analysed separately.
Target condition	Primary: type 1 AMI, defined per the Fourth Universal Definition of MI as: biochemical: rise/fall in cTn with ≥1 value>99th percentile URL. Clinical: ischaemic symptoms, ECG changes or imaging evidence.Secondary: all types of AMI; MACE at 30 days including all-cause mortality and incident AMI.We will also consider data for unstable angina or for a composite of ACS (ACS, comprising AMI and unstable angina), where they are presented.
Reference standard	Laboratory-based troponin testing in hospital will be considered part of the clinical reference standard for the diagnosis of AMI. We will do a cross-tabulation of POC troponin test results vs lab-based troponin test results to evaluate diagnostic agreement. The reference standard for diagnosing AMI will include clinical evaluation, ECG findings and laboratory-based troponin levels as defined by the contemporaneous version of the universal definition of MI and should ideally be adjudicated by at least two independent investigators.A time frame will be applied to identify false negatives, specifically evaluating outcomes within 30 days to capture patients who may not be transported to hospital immediately but later develop AMI.

This table provides a structured overview of the critical components used to assess the accuracy of POC troponin tests, ensuring a thorough and systematic evaluation.

AMI, acute myocardial infarction; cTn, cardiac troponin; MACE, major adverse cardiac events.

#### Clinical effectiveness

To assess the clinical effectiveness of POC troponin tests, with or without clinical decision aids, various outcomes related to patient care in prehospital settings must be evaluated. These outcomes include MACE, healthcare costs and patient satisfaction. MACE will include all-cause mortality, recurrent AMI and revascularisation within 30 days. Healthcare costs will be categorised into direct costs (ambulance use, hospital admissions, ED visits) and indirect costs if reported. Patient outcomes such as satisfaction and decision regret will be extracted when available, using the scales specified in each study. Outcomes will be recorded as categorical or continuous variables based on the original data. The criteria for evaluating clinical effectiveness, such as population, intervention, comparator and outcomes, are detailed in table 2.

**Table 2:** ICO 2: clinical effectiveness

Population	Adult patients (aged 18 years and over) receiving an emergency ambulance response for pain or discomfort in the chest, epigastrium, neck, jaw, arm(s) and/or throat that the treating paramedic suspects may have been caused by AMI.Exclusions: patients with ST-elevation myocardial infarction.
Intervention	cTn point-of-care test +/− a clinical decision aid such as the HEART score or T-MACS decision aid, used to identify ‘very low risk’ patients who may avoid conveyance to hospital.We will include data for all cTn tests, but we will run subgroup analyses for high-sensitivity and contemporary troponin assays.Thresholds for troponin test positivity: limit of detection of the assay; 99th percentile upper reference limit.Threshold for decision aid positivity: ‘very low risk’ for T-MACS; ‘low risk’ for HEART score; the lowest risk group for all other decision aids analysed.Blood samples should have been drawn in the prehospital environment. Ideally, the tests should have been run by paramedics in the prehospital environment using whole blood samples.
Comparator	Usual care with no POC troponin testing, involving routine transport to hospital on paramedic suspicion of AMI.
Outcomes	Primary: MACE within 30 days, including death and AMI.Secondary: healthcare costs; healthcare resource utilisation including subsequent emergency ambulance calls and ED visits; time to discharge; proportion of patients conveyed to hospital; proportion of patients admitted to hospital; missed diagnoses of AMI; any-patient relevant outcome, such as patient satisfaction and decision regret.

This table provides a clear framework for analysing the impact of POC troponin tests on patient management and healthcare resource utilisation in EMS.

AMI, acute myocardial infarction; MACE, major adverse cardiac events.

### Reference standard for type 1 acute myocardial infarction

The diagnosis of type 1 AMI must be adjudicated by investigators using the Fourth Universal Definition of MI.^8^ Laboratory-based troponin testing must be performed in accordance with ESC/ACC guidelines, with serial measurements demonstrating a rise/fall pattern. Studies lacking independent adjudication or using non-validated criteria will be excluded.

### Searches

A single researcher will conduct the searches using a predefined search strategy developed in collaboration with an information specialist experienced in systematic reviews. The search strategy will be agreed on by the research team to ensure consistency and comprehensiveness. A draft of the search strategy will be developed at the protocol phase, although it is not a requirement for PROSPERO registration.

We will search MEDLINE, EMBASE and CINAHL for studies on adult patients with symptoms compatible with AMI in prehospital settings. The full search strategy for all databases is provided in online supplemental file 1. We will only include full-text papers published in English after 2000. All relevant studies will be considered for inclusion. Translation services will be sought for non-English studies where possible, and studies will only be excluded if suitable translation cannot be obtained. Studies published before 2000 were excluded due to the significant evolution of troponin assays after this period, particularly the introduction of high-sensitivity assays. Including older studies would introduce outdated diagnostic methods that are not reflective of contemporary prehospital care practices, potentially skewing the review’*s* findings. The date cut-off of 2000 is chosen because significant advancements in cTn testing occurred after this year. Animal studies and papers published before 2000 will be excluded.

In addition to these databases, we will search grey literature and trial registries to ensure a comprehensive inclusion of studies, including unpublished or ongoing research, to reduce the risk of publication bias.

- OpenGrey and British Library EthOS will be searched for relevant reports, theses and other non-commercially published literature.
- ClinicalTrials.gov, WHO ICTRP and the EU Clinical Trials Register will be searched for ongoing or completed trials related to point-of-care troponin testing that have not yet been published.
- PROSPERO will be searched to identify any ongoing or planned systematic reviews relevant to this topic to avoid duplication and capture emerging evidence.

### Types of study to be included

- To assess diagnostic test accuracy, we will include prospective and retrospective diagnostic test accuracy studies, both comparative and non-comparative. Two-gate or ‘diagnostic case-control’ studies will be excluded due to the high risk of bias.
- To assess clinical effectiveness, we will include randomised controlled trials (RCTs) and any non-randomised comparative studies (eg, before-and-after studies and implementation studies).

To manage screening and data extraction, we will use Rayyan software. This tool will facilitate duplicate removal, title and abstract screening, full-text review and data extraction. Two researchers will screen studies independently, and any disagreements will be resolved by a third reviewer.

### Data management

Rayyan software will be used to manage the screening process for titles and abstracts. Data extraction will be performed using standardised spreadsheets designed specifically for this review. Two reviewers will independently screen titles, abstracts and full texts. Disagreements will be resolved by a third reviewer. Extracted data will be stored in standardised spreadsheets, ensuring clarity and consistency.

All data will be securely stored on the university’s server with regular backups. For long-term access, datasets and relevant materials will be deposited in Zenodo, where they will be available for a minimum of 5 years. The corresponding author will handle any additional data requests postpublication, ensuring transparency and access.

### Data extraction (selection and coding)

Titles and abstracts and full texts of relevant records will be screened by two investigators acting independently of each other. Disagreements and discrepancies between investigators over the eligibility of studies and biases will be resolved through discussion with third investigators.

A standardised extraction form will be used to extract data in a spreadsheet format by one of two investigators. The data extraction form will be piloted on three studies prior to finalising its content.

#### PICTR 1: diagnostic test accuracy

We will extract available data on patient demographics (to include gender, ethnicity, age and comorbidities as well as setting, index test (assay/platform, threshold), details relating to conduct of the index test and reference standard), and all data required to reconstruct 2×2 tables to evaluate diagnostic test accuracy. If the 2×2 table cannot be reconstructed, we will contact the authors of the paper. If the data still cannot be obtained, we will include the data in a narrative synthesis using author-reported metrics of diagnostic accuracy, but the data will be excluded from any meta-analysis. We will extract author-reported data on measures of diagnostic accuracy (sensitivity, specificity, positive predictive value, negative predictive value, positive likelihood ratio, negative likelihood ratio, area under the receiver operating characteristic (ROC) curve, diagnostic ORs and their respective 95% CIs). Each of these metrics adds useful information to address the research question, hence the inclusion of a comprehensive set of metrics around diagnostic test accuracy.

Data extraction will be completed by two investigators acting independently. All data relating to primary evaluation of diagnostic test accuracy (ie, data required to construct 2×2 tables) will be extracted by each of the two investigators. All other data will be extracted by a single investigator and checked by a second investigator.

#### PICO 2: clinical effectiveness

We will extract available data on patient demographics (to include gender, ethnicity, age and comorbidities), the index test (assay/platform, threshold, details of its conduct) and reference standard. We will also extract data relating to each outcome studied, including the number and proportion of patients meeting criteria for each relevant outcome measure and the details of any statistical testing undertaken by the authors. We will extract all data reported on total healthcare costs (from both a healthcare and a societal perspective). Although this systematic review is not designed to specifically evaluate cost-effectiveness, we will extract author-reported data on measures of cost-effectiveness or cost-utility.

This systematic review will include an evaluation of the cost-effectiveness of POC troponin testing in prehospital settings, where available. Economic data will be extracted and assessed using the Consolidated Health Economic Evaluation Reporting Standards checklist, as stated in the PROSPERO registration. Studies reporting on healthcare costs, resource utilisation or cost-effectiveness outcomes will be included in the analysis.

Data extraction will be completed as for PICTR 1.

### Data handling and assumptions

In cases where missing data or inconsistent reporting occurs, the following assumptions and simplifications will be applied:

- Missing diagnostic data will be handled by contacting study authors. If unavailable, the study will be included in a narrative synthesis but excluded from quantitative analysis.
- For studies reporting non-standard diagnostic thresholds, sensitivity analysis will be performed to assess potential impact.
- Missing or inconsistent data on healthcare costs and resource utilisation will be incorporated into the narrative synthesis, and composite outcomes will be simplified to focus on AMI-specific outcomes where possible.

### Risk of bias (quality) assessment

We will assess the methodological quality of the selected studies and risk of bias using appropriate tools for different types of studies.

#### Diagnostic test accuracy studies

This protocol explicitly uses the Quality Assessment of Diagnostic Accuracy Studies version 2 (QUADAS-2) tool to assess the risk of bias in diagnostic accuracy studies, ensuring transparency and methodological rigour. A modified version of QUADAS-2, tailored to our research question, will be employed.^16 17^ The final version of the modified QUADAS-2 tool will be published alongside our findings. Two different reviewers will go through the included paper to assess the risk of bias. Any discrepancies between reviewers will be solved by discussion. Initial scoping of the literature suggests that we are unlikely to find comparative accuracy studies. However, if we should identify comparative accuracy studies, we will use a modified version of the Quality Assessment of Diagnostic Accuracy Studies version 2 (QUADAS-C) tool for quality assessment.^17^

#### Effectiveness studies

For clinical effectiveness studies, we will use the Cochrane Risk of Bias 2 tool for RCTs and the Risk of Bias in Non-randomised Studies of Interventions (ROBINS-I) tool for non-randomised comparative studies. These tools will help assess bias in study design, conduct and reporting. Two different reviewers will go through the included papers to assess the risk of bias. Any discrepancies between reviewers will be solved by discussion.^18^

When using the ROBINS-I tool, the following confounders will be considered: age, gender and ethnicity of participants; the proportion of patients with ECG ischaemia; a previous history of MI or coronary intervention and the prevalence of AMI. The following co-interventions will also be considered as potential sources of bias: shared decision-making and implementation of a new care pathway that accounts for factors other than the test or decision aid being evaluated.

Additional confounders and/or co-interventions may be identified when reviewing individual studies. Each of the two reviewers will indicate potential confounders. Each potential confounder/co-intervention will then be reviewed by the study team. The study team will make a final decision about the inclusion of a potential confounder/co-intervention when assessing the risk of bias, based on its perceived potential impact. A majority of the study team must agree for an additional confounder/co-intervention to be included.

The risk of bias tools for intervention studies will be unmodified, though the study team will issue guidance notes to clarify key issues (such as determining the appropriate means to measure outcomes) prior to commencing analysis.

### Strategy for data synthesis

#### Diagnostic test accuracy

All statistical analyses and methods for data synthesis have been predefined in this protocol to ensure methodological rigour and reduce bias. For diagnostic accuracy, we will use hierarchical models, applying a bivariate model when sufficient data for a common troponin threshold exist, and HSROC methods otherwise. Clinical heterogeneity will be assessed using predefined criteria including setting, patient demographics and assay type. We will use forest plots to demonstrate paired sensitivity and specificity for each test/decision aid evaluated, and we will plot the data in a ROC space.

To assess heterogeneity, we will first visually examine the ROC space for evidence of potential threshold effects. Clinical heterogeneity will be assessed qualitatively by the investigators, based on study characteristics including the setting, patient group, prehospital system, troponin assay, clinical decision aids applied and other factors felt to be of relevance. The study team will review these data to determine whether proceeding to meta-analysis is appropriate.

If we proceed to meta-analysis, we will use hierarchical models, and we will apply a random effects model. Should there be sufficient data for the use of a common troponin threshold with the same assay, we will meta-analyse the data using a bivariate model.^19^ If there are insufficient data using a common threshold, we will use HSROC methods to estimate sensitivity and specificity at thresholds to optimise sensitivity. We will aim for the maximum sensitivity that achieves at least 98%, or the highest sensitivity that may be achieved, while considering acceptable levels of specificity.

If meta-analysis is not possible, we will undertake a narrative synthesis of the search findings and the included studies. We will perform statistical analyses using Stata (using the METANDI, MAR and/or MIDAS commands) and/or R.

#### Clinical effectiveness

The synthesis should begin with descriptive summaries of patient demographics, settings, troponin assays and clinical decision aids. Heterogeneity will be assessed using the I² statistic and Cochran’s Q test, with a random effects model employed for meta-analysis if appropriate.

Subgroup analyses will explore heterogeneity sources, including gender, ethnicity, type of troponin assay and clinical decision aid, alongside sensitivity analyses to ensure robustness. Continuous outcomes will be analysed using weighted mean differences or standardised mean differences, and dichotomous outcomes will be assessed using pooled risk ratios.

Statistical analyses will be conducted using Stata and/or R. If meta-analysis is not feasible, a narrative synthesis will describe the study findings, focusing on effect direction, magnitude and consistency.

### Limitations

This review may be limited by the availability of robust studies on POC troponin testing in prehospital care.^14^ Although we will seek translation for non-English studies, some may still be excluded if suitable translation is not possible. The fast pace of technological advancements in troponin assays means older studies may not reflect current practice, while newer studies may still be scarce.^6^ Differences in prehospital systems, troponin assays and decision aids across studies could introduce variability, making it harder to combine findings.^18^ If data are incomplete or inconsistent, we may need to rely on narrative summaries rather than meta-analysis.

#### Meta-bias

We will assess for potential biases across studies, including publication bias and selective reporting. Funnel plots will be used if 10 or more studies are available for meta-analysis. Funnel plot asymmetry will be examined visually, and statistical tests such as Egger’s or Begg’s will be employed to detect any bias. Should asymmetry be found, we will explore causes such as small study effects and report on how these may influence the overall findings.

#### Confidence in cumulative evidence

We will assess the strength of the evidence using the Grading of Recommendations Assessment, Development and Evaluation framework, which considers factors like study quality, consistency and precision. Each outcome will be rated as high, moderate, low or very low quality. We will present the findings in a summary table, showing the level of confidence in the main outcomes, including diagnostic accuracy and clinical impact.

### Analysis of subgroups or subsets

Subgroups of interest a priori include gender, ethnicity, troponin assay, decision aid used and setting in which testing occurs. If the data permit meaningful subgroup analyses, we will proceed to analyse the data based on these subgroups.

### Patient and public involvement statement

No patients or members of the public were involved in the design or conduct of this study. Results will be disseminated through peer-reviewed publication and conference presentations.

## Ethics and dissemination

As this study involves secondary analysis of published data, ethics approval is not required. Results will be shared with EMS agencies, clinical guideline developers and academic audiences.

### Proposed amendments

At the time of protocol registration, no amendments have been made. Should any amendments be necessary during the review process, they will be documented in table 3.

**Table 3:** Amendments

Date of amendment	Section of protocol	Original protocol text	Amended protocol text	Rationale for amendment
N/A	N/A	N/A	N/A	N/A

Any amendments will be recorded in the table, detailing the nature of the change, the affected section, the original and amended text and the rationale for the revision.

## Supplementary material

10.1136/bmjopen-2024-094390 online supplemental file 1

10.1136/bmjopen-2024-094390 online supplemental file 2
