# Supplementary material for: Point-of-care troponin tests to rule out acute myocardial infarction in the prehospital environment: a protocol for a systematic review and meta-analysis
Source: BMJ Open. 2025 May 2;15(5):e094390. doi: 10.1136/bmjopen-2024-094390 (PMC12049932; doi:10.1136/bmjopen-2024-094390)
Supplement: online supplemental file 1 [file bmjopen-15-5-s001.docx]

Supplementary File 1: Ovid MEDLINE (R) ALL Search Strategy

Database: Ovid MEDLINE (R) ALL

Coverage: 1946 to October 04, 2024

Search Date: October 09, 2024

Search Conducted by: Sarah Dawson

Objective: To identify studies related to prehospital point-of-care troponin testing for acute myocardial infarction diagnosis.

Ovid MEDLINE(R) ALL <1946 to October 04, 2024>

1     ambulances/ or air ambulances/      10362

2     emergency responders/   1310

3     paramedics/ or emergency medical technicians/   6282

4     emergency medical services/   50489

5     emergency medical dispatch/   198

6     ambulanc*.mp.     19537

7     ((ambulatory or mobile) adj emergenc*).tw,kf.   439

8     ((helicopter? and emergenc*) or aeromedic* or aero-medic* or airmedic* or air medic*).tw,kf.    4285

9     (emergenc* adj2 (craft? or crew or dispatch* or personnel or responder? or technician? or transport* or vehicle? or helicopter?)).tw,kf.  6963

10    (emergency adj3 (setting or service*)).tw,kf.   34913

11    (prehospital* or pre-hospital* or out-of-hospital).tw,kf.   37585

12    (paramed* or para-med*).tw,kf.      15740

13    ((first or frontline or front line) adj responder?).tw,kf.  3860

14    or/1-13     123667

15    troponin i/ or troponin t/    15623

16    (cardiac trop* or cTn or cTnI or cTnT or hscTn or hscTnI or hscTnT or Tropoinin-I or Troponin-T or TnI or TnT or hsTnI or hsTnT).tw,kf.   28727

17    15 or 16    32796

18    14 and 17   356

19    troponin.mp.      38349

20    exp Cardiovascular Diseases/  2821838

21    exp Myocardial Ischemia/      484241

22    exp Chest Pain/   58452

23    (chest adj3 pain*).tw,kf.     44785

24    (angina* or cardio* or cardiac* or coronary or heart or myocardi*).mp.  2816114

25    (ACS or NSTE-ACS).tw,kf.      31997

26    (AMI or NSTEMI).tw,kf.  28315

27    or/20-26    4108558

28    19 and 27   33571

29    14 and 28   546

30    18 or 29    566

31    (rule out and (heart attack* or myocardial infarct* or AMI or NSTEMI)).tw,kf. 1032

32    14 and 31   67

33    30 or 32    594

34    (Radiomet* and (AQT90* or AQT-90*)).mp.   17

35    ((AQT90* or AQT-90*) adj3 Flex*).mp.      24

36    (Siemens* and (Atellica* or VTLi*)).mp.   119

37    (Atellica* adj3 VTLi*).mp.    16

38    (Roche* and (Cobas* or h232*)).mp.  2087

39    ("cobas h232*" or "cobas h 232*" or "cobash232*").mp. 16

40    (Abbott* and i-Stat*).mp.     96

41    (i-stat or i-statR or i-statTM or (i-STAT* and (CG4? or EC8?))).mp.     337

42    T-MACS*.mp. 26

43    ((Alere* or Biosite* or Quidel*) and Triage*).mp.     140

44    (Triage MeterPlus* or Triage MetrePlus* or Triage Met* Plus*).mp. 4

45    (Triage MeterPro* or Triage MetrePro* or Triage Met* Pro*).mp.    4

46    (Biosite* and (Triage* or Meter* or Metre*)).mp.      98

47    (Siemens* and Stratus*).mp.   8

48    Stratus CS*.mp.   50

49    (Samsung* and Lab*).mp. 121

50    (LabGeo* or Lab-GEO*).mp.     10

51    ((Phillips* or Philips*) and (Minicare* or Mini-care*)).mp. 2

52    ("LSI Medicence" or PATHFAST*).mp.  58

53    (Response Biomedical and RAMP*).mp. 6

54    (Spectral* and Cardiac Status*).mp. 6

55    (Roche* and (cardiac* or "Trop T")).mp.   861

56    (CARDIAC Trop T Sensitive* or Cardiac Reader*).mp.    17

57    (Alere* and Cardio*).mp.      13

58    (Cardio2 or Cardio-2 or Cardio3 or Cardio-3).mp.      21

59    Cardio? panel.mp. 7

60    or/34-59    3758

61    14 and 60   81

62    33 or 61    649

63    limit 62 to yr="2000 -Current"      617

64    limit 63 to english language  579

65    ((neonat* or infant* or child* or p?ediatri*) not adult*).ti.     1425035

66    64 not 65   571

67    exp animals/ not humans/      5265712

68    66 not 67   564
