# Supplementary material for: Point-of-care troponin tests to rule out acute myocardial infarction in the prehospital environment: a protocol for a systematic review and meta-analysis
Source: BMJ Open. 2025 May 2;15(5):e094390. doi: 10.1136/bmjopen-2024-094390 (PMC12049932; doi:10.1136/bmjopen-2024-094390)
Supplement: Supplementary file 3 [file bmjopen-2024-094390.reviewer_comments.pdf]

PEER REVIEW HISTORY

BMJ Open publishes all reviews undertaken for accepted manuscripts. Reviewers are asked to complete a checklist review form and are provided with free text boxes to elaborate on their assessment. These free text comments are reproduced below.

ARTICLE DETAILS

Title (Provisional)

Point of care troponin tests to rule out acute myocardial infarction in the prehospital environment a protocol for a systematic review and meta- analysis.

Authors

Albaqami, Bader; Dinnes, Jac; Moore, Theresa HM; Kirby, Kim; Carley, Simon D; Aloufi, Mohammad; Alqurashi, Naif; Alghamdi, Abdulrhman; Alsuwais, Sara; Dawson, Sarah; Body, Richard

---

VERSION 1 - REVIEW

**Reviewer**	**1**
**Name**	**Defilippis, Andrew P.**
**Affiliation**	**Vanderbilt University Medical Center**
**Date**	**16-Dec-2024**
**COI**	**None**

• The introduction clearly describes the relevance of the issue they propose to study—the diagnosis of acute myocardial infarction. However, in the reviewer’s opinion, the introduction lacks a robust description of the diagnostic challenges of acute myocardial infarction. The introduction should note how the study design will account for the fact that, by definition, high sensitivity cardiac troponin (cTn) assays have a limit of detection that is low enough to allow for quantification of circulating cTn in > 50% of healthy individuals. It should be noted that cTn detects myocardial injury and that myocardial infarction is only one type of myocardial injury—criteria for myocardial infarction require more than evidence of myocardial injury. Furthermore, while the stated aim of this meta-analysis is to evaluate the ability to diagnose type 1 MI, there are multiple types of MI per the Universal Definition of MI and cTn does not differentiate between these types of MI. Additionally, although chest pain is the single most common symptom identified in acute MI patients, the majority of acute MI patients do not report chest pain as their primary symptom at the time of presentation.

• Clarification on the existence of a high-sensitivity POC cTn assay.

• The introduction fails to describe how the results of the proposed meta-analysis will change current practice.

• Clarity is needed for the diagnosis of the target condition “type 1 AMI”. What are the acceptable criteria for determining this diagnosis?

• While treatment guidelines exist for type I NSTEMI, e.g., heparin drip and other interventions these intervention have the potential for significant harm and no clearly demonstrated benefit in non-type 1 MI myocardial injuries.

• Originality: In the introduction, it is acknowledged that this meta-analysis is an updated review on the Alghamdi et al review (which identified promise but gaps in the accuracy of the tests) to account for rapid advancements in technology after the year 2000.

• Research question: What is a protocol for assessing the diagnostic accuracy, clinical utility, and safety of cardiac troponin point of care tests, with or without clinical decision aids, for “ruling out” AMI in adults presenting with symptoms of cardiac chest pain to EMS? The objectives should clarify that this is a protocol and not the meta-analysis itself. For example, the “Objective” section of the abstract is misleading in its current form—the objective of this work is to describe the methodology for performing a meta-analysis that will answer the research question noted above.

• Clarity is needed throughout the manuscript about the objective of this manuscript. For example, point one under Article Summary: “This systematic review will build on previous work by…………” is misleading in this reviewer's opinion and should read “This proposed meta-analysis study design aims to ……………..”

• This topic is of importance to the general medical community, particularly emergency providers, cardiologists, hospital administrators, and EMS medical directors, as it can give insight into the efficacy and safety of utilizing cardiac troponin tests with or without clinical decision aids.

• The study design to answer each part of the research question is described. It starts with diagnostic test accuracy as described in PICTR 1. Data were included for all POC cardiac troponin tests, but subgroup analyses were performed for high-sensitivity versus conventional assays. The paper then describes the study design for clinical effectiveness as described in PICO 2. The authors describe the search process utilizing MEDLINE, EMBASE, and CINAHL in addition to grey literature, trial registries, and planned systemic reviews (OpenGrey and British Library EthOS, ClinicalTrials.gov, WHO ICTRP, EU Clinical Trials Register, and PROSPERO).

• Authors have a good plan for missing data or inconsistent reporting.

• I am less familiar with risk of bias assessment, but the QUADAS-2 is recommended for use in systematic reviews to evaluate the risk of bias and applicability of primary diagnostic accuracy studies. Looking into Cochrane Risk of Bias 2 and ROBINS-I, they seem to be a good choice for RCTs and non-randomized comparative studies, respectively.

• Lastly, their strategy for data synthesis appears to be appropriate for diagnostic accuracy (Forest plots) and for clinical effectiveness (table 1 summary, followed by subgroup analyses for heterogeneity).

Reviewer **2**

Name **Hernández-Vásquez, Akram**

Affiliation **Universidad San Ignacio de Loyola, Vicerrectorado de Investigación, Centro de Excelencia en Investigaciones Económicas y Sociales en Salud**

Date **27-Dec-2024**

COI **None**

---

1. The phrase "recent further development using clinical decision aids" could be clarified to explain how tools like the HEART score or T-MACS integrate troponin data and other clinical parameters.

2. The numbering of citations appears inconsistent and out of sequence (e.g., reference 7 is cited before 6).

3. The introduction briefly mentions delays associated with hospital-based troponin testing but could elaborate more on the practical implications (e.g., prolonged EMS time, increased healthcare costs).

4. The statement on gaps in diagnostic accuracy from the Alghamdi et al. review is significant but underexplored.

5. While the introduction discusses diagnostic pathways, it does not address the impact of POC troponin testing on patient-centered outcomes (e.g., mortality, morbidity, or time to treatment).

6. While the outcomes (e.g., MACE, healthcare costs) are mentioned, there is limited detail on how these will be measured or categorized.

7. Justify the exclusion of non-English studies or consider including studies in other major languages with translation support.

8. The exclusion of papers published before 2000 is justified by advancements in technology but may overlook historical studies relevant for understanding diagnostic test evolution. Clarify how these exclusions impact the review's scope and address potential biases introduced by this cut-off.

9. While the objectives and methods are well-structured, the practical implications for EMS systems and patient management are not clearly articulated. Add a statement on how findings will influence EMS protocols or patient outcomes.

10. Stating that the analysis plan will be "finalized after data extraction" does not meet methodological standards for protocols, as the analysis should be predefined before data collection begins to minimize biases.

11. There are significant discrepancies between the protocol registered in PROSPERO and the manuscript. For instance, the protocol references the use of the Consolidated Health Economic Evaluation Reporting Standards (CHEERS) checklist, yet the manuscript does not mention any economic evaluations.

12. The statement by the authors that "Rayyan software will be used to manage screening and data extraction" is incorrect, as Rayyan is not designed for data extraction. It is a tool specifically intended for managing and streamlining the screening process of systematic reviews. Data extraction requires other dedicated tools or manually designed templates.

13. The authors' statement that "Two researchers will conduct the searches independently, following the same predefined search strategy" reflects a misunderstanding of best practices for systematic review searches. It is not feasible for two researchers to independently perform identical searches, as search strategies require consistency to ensure reproducibility. Moreover, it is highly recommended that a librarian or information specialist, with expertise in systematic search strategies, be involved in the development and execution of the search to maximize comprehensiveness and accuracy.

14. It would be important for the authors to include at least a section discussing the limitations of their study. Addressing potential methodological or practical constraints would enhance the transparency and reliability of the findings and provide a balanced perspective on the study's implications.

15. It would be important for the authors to explicitly state in the methods section that the study aligns with PRISMA-P guidelines, rather than only mentioning it in the abstract. This ensures clarity and reinforces the adherence to established standards for systematic review protocols throughout the manuscript.

16. It would be important for the authors to review whether it is appropriate to use a guideline designed for reviews of interventions when the focus of the study is on diagnostic methods. Similarly, they must ensure compliance with all PRISMA-P elements and include the completed PRISMA-P checklist as supplementary material.

VERSION 1 - AUTHOR RESPONSE

Reviewer 1 Comments:

1. **Diagnostic Challenges of Acute Myocardial Infarction:**
    - We have expanded the Introduction to include a more robust discussion of the diagnostic challenges of AMI. Specifically, we have clarified that high sensitivity cTn assays detect MI, which is not exclusive to AMI. We have also noted that the diagnosis of AMI requires clinical evidence of ischemia, as outlined in the Fourth Universal Definition of MI. Additionally, we have addressed the fact that chest pain, while common, is not the only symptom of AMI, particularly in women, older adults, and individuals with diabetes.

2. **Clarification on High-Sensitivity POC cTn Assay:**
    - We have clarified in the Introduction that high-sensitivity POC cTn assays, such as the Roche cobas h232 troponin T and Siemens Atellica VTLi troponin I, are validated for clinical use and meet ESC/ACC criteria for high sensitivity. These assays allow for quantification of cTn concentrations as low as 3–5 ng/L, making them suitable for prehospital use.

3. **Impact on Current Practice:**
    - We have added a discussion in the Introduction on how the results of this meta-analysis could change current practice. Specifically, we highlight that demonstrating the reliability of POC troponin assays could allow paramedics to safely avoid transporting low-risk patients, expedite treatment for those requiring urgent care, and reduce unnecessary heparin use in non-ischemic cases. These improvements could enhance ambulance service efficiency, lower healthcare costs, and maintain patient safety.

4. **Clarity on Diagnosis of Type 1 AMI:**
    - We have clarified in the Methods section that the diagnosis of type 1 AMI must be adjudicated using the Fourth Universal Definition of MI, which includes biochemical evidence (rise/fall in cTn with at least one value above the 99th percentile upper reference limit) and clinical evidence (ischemic symptoms, ECG changes, or imaging evidence). Studies lacking independent adjudication or using non-validated criteria will be excluded.

5. **Potential Harm of Interventions in Non-Type 1 MI:**
    - We have added a note in the Introduction regarding the potential harm of interventions such as heparin in non-type 1 MI cases. This highlights the importance of accurately differentiating between type 1 and non-type 1 MI to avoid unnecessary and potentially harmful treatments.

6. **Clarity on Objective:**
    - We have revised the Objective section in the Abstract and Introduction to clarify that this is a protocol for a systematic review and meta-analysis, not the meta-analysis itself. The objective now reads: "To present a protocol for a systematic review and meta-analysis that will assess the diagnostic accuracy, clinical utility, and safety of POC troponin tests, with or without clinical decision aids, for ruling out AMI in adults presenting with cardiac chest pain to emergency ambulance services in prehospital settings."

7. **Clarity on Study Design:**

- We have revised the Article Summary to clarify that this is a proposed meta-analysis study design. The text now reads: "This proposed meta-analysis study design aims to build on previous work by evaluating the diagnostic accuracy, clinical utility, and safety of POC troponin tests in prehospital settings, providing an updated synthesis of evidence to guide clinical practice."

8. **Risk of Bias Assessment:**
   - We have retained the use of QUADAS-2 for diagnostic accuracy studies, Cochrane Risk of Bias 2for randomized controlled trials, and ROBINS-I for non-randomized comparative studies, as these tools are appropriate for the study design and ensure methodological rigor.

9. **Strategy for Data Synthesis:**
   - We have retained the planned use of hierarchical models for diagnostic accuracy and random-effects models for clinical effectiveness, as outlined in the Methods section. Forest plots and ROC space analysis will be used to assess diagnostic accuracy, while subgroup analyses will explore heterogeneity.

Reviewer 2 Comments:

1. **Clarification on Clinical Decision Aids:**
   - We have expanded the discussion of clinical decision aids in the Introduction to explain how tools like the HEART score and T-MACS integrate troponin levels with clinical factors such as patient history, symptoms, and ECG findings. This integration helps paramedics assess AMI risk more accurately and improve decision-making in prehospital settings.

2. **Citation Numbering:**
   - We have reviewed and corrected the citation numbering throughout the manuscript to ensure consistency and proper sequencing.

3. **Practical Implications of Delays:**
   - We have expanded the discussion in the Introduction on the practical implications of delays associated with hospital-based troponin testing, including prolonged EMS time, increased healthcare costs, and the strain on healthcare resources.

4. **Exploration of Diagnostic Accuracy Gaps:**
   - We have further explored the gaps in diagnostic accuracy identified by Alghamdi et al. in the Introduction, emphasising the need for more precise assays, uniform cutoff standards, and rigorously designed studies to confirm the reliability of POC troponin tests in prehospital settings.

5. **Impact on Patient-Centered Outcomes:**

- We have added a discussion in the Introduction on the potential impact of POC troponin testing on patient-centered outcomes, including mortality, morbidity, and time to treatment. This highlights the clinical relevance of the study.

6. **Measurement of Outcomes:**
    - We have provided more detail in the Methods section on how outcomes such as MACE and healthcare costs will be measured and categorised. Specifically, MACE will include all-cause mortality, recurrent AMI, and revascularisation within 30 days, while healthcare costs will be categorized into direct and indirect costs.

7. **Justification for Excluding Non-English Studies:**
    - We have clarified in the Methods section that non-English studies will be included if translation support is available. Studies will only be excluded if suitable translation cannot be obtained.

8. **Exclusion of Pre-2000 Studies:**
    - We have justified the exclusion of studies published before 2000 in the Methods section, noting that significant advancements in cardiac troponin testing occurred after this period. Including older studies would introduce outdated diagnostic methods that are not reflective of contemporary prehospital care practices.

9. **Practical Implications for EMS Systems:**
    - We have added a statement in the Introduction on how the findings of this review could influence EMS protocols and patient outcomes, particularly in terms of reducing unnecessary hospital admissions and expediting treatment for high-risk patients.

10. **Predefined Analysis Plan:**
    - We have revised the Methods section to clarify that the analysis plan is predefined and will not be finalised after data extraction. This ensures methodological rigor and minimises bias.

11. **Discrepancies with PROSPERO Protocol:**
    - We have reviewed the manuscript to ensure consistency with the PROSPERO protocol, including the use of the CHEERS checklist for economic evaluations.

12. **Clarification on Rayyan Software:**
    - We have clarified in the Methods section that Rayyan software will be used for screening and managing references, while data extraction will be performed using standardised spreadsheets. This ensures accurate data management.

13. **Search Strategy Execution:**

- We have revised the Methods section to clarify that a single researcher, in collaboration with an information specialist, will conduct the searches to ensure consistency and reproducibility.

14. **Study Limitations:**
    - We have added a Limitations section in the Discussion to address potential methodological and practical constraints, enhancing the transparency and reliability of the findings.

15. **PRISMA-P Compliance:**
    - We have explicitly stated in the Methods section that the study aligns with PRISMA-P guidelines and have included the completed PRISMA-P checklist as supplementary material.

16. **Guideline for Diagnostic Methods:**
    - We have ensured that the study design aligns with PRISMA-P guidelines for diagnostic test accuracy reviews, and we have included the completed PRISMA-P checklist as supplementary material.

VERSION 2 - REVIEW

Reviewer **2**

Name **Hernández-Vásquez, Akram**

Affiliation **Universidad San Ignacio de Loyola, Vicerrectorado de Investigación, Centro de Excelencia en Investigaciones Económicas y Sociales en Salud**

Date **07-Apr-2025**

COI

The authors failed to properly document and address the reviewers' comments. It appears they lack familiarity with the peer-review process. It is inappropriate to modify specific reviewer observations and replace them with vague, generalized statements open to subjective interpretation. In a response letter, the correct approach is to: (1) quote the reviewer’s comment verbatim, (2) provide a direct response to it, and (3) clearly specify and indicate the corresponding revisions made in the manuscript. This structured process is not followed in the current response letter, making it impossible to cross-reference the authors’ replies and claimed changes with the manuscript’s content.

Reviewers' time is valuable and they are not compensated for it.
